# Energy-Aware Management in Multi-UAV Deployments: Modelling and Strategies

**DOI:** 10.3390/s20102791

**Published:** 2020-05-14

**Authors:** Victor Sanchez-Aguero, Francisco Valera, Ivan Vidal, Christian Tipantuña, Xavier Hesselbach

**Affiliations:** 1 IMDEA Networks Institute, Avda. del Mar Mediterráneo, 22, 28918 Madrid, Spain; 2 Departamento de Ingeniería Telemática, Universidad Carlos III de Madrid, Avda. Universidad, 30, 28911 Leganés, Madrid, Spain; fvalera@it.uc3m.es (F.V.); ividal@it.uc3m.es (I.V.); 3 Department of Network Engineering, Universitat Politècnica de Catalunya, Calle Jordi Girona 1-3, E-08034 Barcelona, Spain; christian.jose.tipantuna@upc.edu (C.T.); xavier.hesselbach@upc.edu (X.H.); 4 Escuela Politécnica Nacional, Ecuador, Avda. Ladrón de Guevara, E11-253 Quito, Ecuador

**Keywords:** UAV, UAV fleet, UAV swarm, energy consumption, self-organization, algorithms, optimization, UAV replacement

## Abstract

Nowadays, Unmanned Aerial Vehicles (UAV) are frequently present in the civilian environment. However, proper implementations of different solutions based on these aircraft still face important challenges. This article deals with multi-UAV systems, forming aerial networks, mainly employed to provide Internet connectivity and different network services to ground users. However, the mission duration (hours) is longer than the limited UAVs’ battery life-time (minutes). This paper introduces the UAV replacement procedure as a way to guarantee ground users’ connectivity over time. This article also formulates the practical UAV replacements problem in moderately large multi-UAV swarms and proves it to be an NP-hard problem in which an optimal solution has exponential complexity. In this regard, the main objective of this article is to evaluate the suitability of heuristic approaches for different scenarios. This paper proposes betweenness centrality heuristic algorithm (BETA), a graph theory-based heuristic algorithm. BETA not only generates solutions close to the optimal (even with 99% similarity to the exact result) but also improves two ground-truth solutions, especially in low-resource scenarios.

## 1. Introduction

The unstoppable growth of the Unmanned Aerial Vehicles (UAVs) (commonly known as drones) ecosystem during these last years, has been proven to be just the beginning of a near-future global phenomenon. The US Federal Aviation Administration predicts [1] that UAVs providing commercial services will triple over the next five years, and will overtake consumer off-the-shelf UAVs by the year 2024. UAVs will grow eightfold over the next decade and will become the largest segment of the civilian market.

The utilization of multi-UAV systems, because of their rapid deployment, mobility, and flexibility, has recently attracted attention to support/extend the fifth-generation cellular network technology (5G) in extraordinary situations (e.g., massified events, natural disasters, infrastructure failures). The 5G networks will certainly bring faster uploading and downloading speeds in combination with a dramatic decrease of the network latency. However, in exceptional or emergency circumstances, the deployment of 5G terrestrial infrastructure may not be economically viable. In addition, the deployment times of these extraordinary on-demand 5G network services should meet the Key Performance Indicators [2] (KPI) defined by the 5G-PPP, which states that new deployments must finish within 90 min. Accordingly, it is here where UAVs are expected to play a crucial role. If properly deployed and configured, UAV networks can provide fast-ubiquitous 5G access (which is also within the 5G KPIs) employing wireless communications solutions in a diversity of real-world scenarios.

5G UAV missions, e.g., to complement existing cellular networks in high-density environments, deliver network coverage in hard to reach rural areas (Remote Access Networks), or in IoT scenarios, may require the management of moderately large UAV fleets. UAVs mainly act as aerial communication platforms such as (i) aerial base stations (BS) (to support existing 5G infrastructure in high traffic demand) [3], or (ii) aerial WiFi access points (AP) forming a Flying Ad Hoc Network (FANET) (to create new networks) [4]. So that UAV research area aims to extend the 5G network (where it has no range) or support the existing 5G network (when it is not enough) using radio solutions as payload [5], e.g., 5G or LTE microcells, multi-hop solutions based on commodity WiFi. When compared to terrestrial antennas, aerial units may have some advantages since they can change their altitude with the possibility of avoiding obstacles, including no geographical restriction on the antenna location. However, these advantages turn into crucial design challenges, such as the optimal positioning, the limited flight time, or the optimal trajectories calculation and network planning [6].

In particular, multi-UAV environments may give rise to long-endurance missions that require uninterrupted service provisioning (performing UAV replacements) that are not achievable using a single UAV due to battery capacity constraints (at most around 20 min flight [7]). A UAV replacement means that a UAV that is waiting in the Ground Control Station (GCS) becomes active and goes into the scenario to substitute one of the UAVs that are on service, so as to provide the same functionality. This is only possible by providing a fleet exceeding the number of UAVs that have to be active on service at the same time, i.e., there is a reasonable number of fresh UAVs for replacement.

Nevertheless, developing an appropriate replacement strategy of UAVs, is one of the critical hurdles that have not yet been properly addressed by the research community. The replacement strategy enables to optimize the cost in terms of required aerial infrastructure resources while keeping the provided level of service. To guarantee that long-term (beyond battery life) services can be deployed, some of the UAVs that are at the GCS must be able to successfully replace the UAVs that provide the actual service on the stage whenever necessary (for example, when a UAV has low battery or fails). However, the economic cost of oversizing the fleet is enormous, as these devices commonly have high prices. For this reason, it is necessary to develop a resource optimization mechanism in order to allow intelligent and autonomous UAV systems to be managed with the lowest possible number of UAVs.

**Figure 1:**
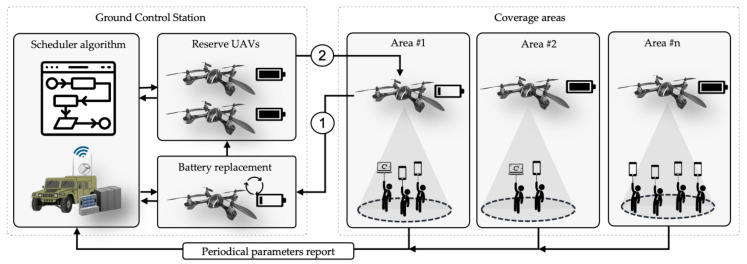
Typical Unmanned Aerial Vehicles (UAV) use case using the proposed methodology.

Figure 1 illustrates a representative use case of UAVs delivering network coverage. As it can be appreciated, some UAVs provide connectivity to several end-users. A Controller entity, located in the GCS, is in charge of scheduling when UAV replacements will take place. Once the replacement procedure is started for a certain UAV, that UAV directly goes back to the GCS to change its battery ① while another one comes back in its place ②. As soon as it has a charged battery installed, it is available again in the replacement pool for other UAVs to be changed when required. By following this methodology, an uninterrupted service may be provided. Reducing the UAV fleet while ensuring a reasonable quality of service is not a straightforward procedure (Further details about the methodology depicted in Figure 1 can be found in Section 4, Section 4.1 and Section 4.4).

This article presents the practical UAV replacement problem and analyses its complexity. Next, different scenarios, as well as the methodology to evaluate the service performance, are presented. A sub-optimal heuristic algorithm is proposed that guarantees the proper modeling and control of a fleet of UAVs that are used to provide Internet connectivity minimizing the fleet size. This algorithm is validated comparing it with the optimal solution, using an improved version of the brute-force search combinatorial algorithm developed in our previous work [8]. Finally, the practical limitations of the proposal are analyzed, while the possible alternatives solutions are considered.

The rest of the article is organized as follows: the related work and background are reviewed in Section 2. Section 3 states the proposed problem and analyses its complexity. Section 4 describes the methodology to operate with large multi-UAV fleets. Then, Section 5 details the suggested scenarios and remarks the results obtained from the simulation. Finally, Section 6 concludes the article and depicts some future research lines.

## 2. Related Work and Background

Due to the versatility of UAVs, these devices are used in a wide variety of fields. The following section introduces the evolution of management strategies used to overcome battery limitations and algorithmic solutions for UAV battery replacements which are the main topics of this article. It also explains how UAVs may complement 5G networks, and wireless communication solutions in large geographical areas using UAV swarms.

Ubiquitous connectivity is one of the current challenges of 5G networks and beyond 5G [6]. UAVs have appeared as a promising solution to provide reliable and flexible wireless communication services for ground users in a wide variety of scenarios [3]. The usage of UAVs promises to provide cost-effective wireless connectivity for devices without infrastructure coverage. Concretely, UAVs are considered as flying BSs for coverage extension and capacity enhancement of the existing 5G cellular networks. In this paper [9], authors explore the use of UAV-BSs to provide coverage during natural disasters. In this work [5], an evolved packet core (EPC) inside a UAV is introduced, to orchestrate the LTE RAN in the presence of multiple BSs. This EPC can also interoperate with commercial BSs as well as commodity user equipment. In [10], the authors provide an overview of UAV-aided networks, introducing the underlying architecture and wireless channel characteristics.

One of the most critical design challenges in multi-UAV systems is the achievement of the all-to-all communication between UAVs, which is necessary for cooperation and collaboration [11,12]. If every UAV is connected to existing network infrastructure such as a GCS, satellite network or base stations, swarm communications can be delivered via this infrastructure. This type of network scheme simplifies some problems that may be associated with UAVs ad hoc networks alternatives, like routing protocols or the distributed control of the network. However, it also brings as a consequence certain limitations such as the expensive equipment (long-range or satellite antennas) and obviously less flexibility since the deployment is fixed to existing infrastructure. An alternative solution is the usage of FANETs. In this type of system, UAVs have several roles, not only as functional devices to provide coverage, gathering sensor data, or video dissemination but also to be used as network relays to connect all UAVs through the UAV network itself. Commonly, only one (or a few) UAV (also known as backbone UAV) are required to be connected to the fixed infrastructure (GCS). The backbone UAV is generally equipped with two radios: (i) low-power radio (WiFi or Bluetooth, for instance) is used for communication between the UAVs and (ii) high power long-range radio to communicate with the GCS [13]. It is common to find quite a few examples of research works that use FANETs to support 5G networks [14]. For instance, Reference [4] extends a 5G network slice for video monitoring with a FANET composed of small low-altitude UAVs with multi-access edge computing (MEC) facilities to allow high-speed transmission.

Although the development of UAV networks is receiving significant attention from the research community, some challenges must be solved before their proper deployment and consolidation. One of them is their limited battery capacity since normally a UAV source power mainly depends on small batteries (we are considering in this article small rotary-wing UAVs and not big fixed-wing UAVs with fuel engines). Consequently, these SUAVs (Small UAVs) are hardware-constrained devices that cannot be too heavy or carry heavy payloads. Besides, to the power consumption of the flight engines, it is essential to consider the additional energy required by onboarded computers, that may not be carrying their own external batteries and in case they were, extra weight would be added to the system. As a consequence, we find that the useful lifetime of a UAV system is undoubtedly limited by these restrictions. Different research works propose solutions to provide uninterrupted service on long endurance missions and overcome the reduced-battery challenge. For instance, Ref. [15] presents an algorithm to offer continuous structural inspection services using UAVs not only through simulation results, but also by using an implementation. In this solution, authors replace a UAV unit before its battery is drained. The replacement algorithm employed in this article will be used later in this paper to compare and contextualize the proposed solution. The modification of their methodology (since in this particular case, authors work with a single-UAV system while the proposed scenarios require moderately large fleets of UAVs) is explained and detailed in Section 5.2.2. In [16], authors consider UAV replacement (among other possible alternatives, such as refueling [17] or recharging) to maintain total surveillance of an area perimeter. Additionally, some articles propose an automatic battery replacement [18,19,20]. They offer a GCS capable of swapping UAV batteries without human interaction. Ground task automation not only reduces human interaction but also increases the multi-UAV system operation area, improving the coverage and enabling operation in hazardous environments. This trend makes us choose battery replacement as the preferred option in the solution proposed in this paper. Battery price is considerably lower than the cost of a UAV, and the time to replace the battery is remarkably shorter than the time to recharge it. Moreover, thanks to these studies and their practical experimentation, we use these results as input for our scheduling algorithms to provide accuracy to the design of UAV replacement strategies. Diverse works attempt to solve the limited battery life problem which is inherent to current SUAVs by proposing diverse alternatives. In [21], it is considered that the UAVs land to provide service (if possible and secure operation). The work in [22] summarizes different techniques to prolong the UAV operation time from Battery dumping [23] to Photovoltaic arrays [24,25]. Some other additional techniques have been proposed like wireless charging using lasers is in [26].

The optimization field, to improve the restricted communication performance of UAV networks while using the minimum amount of physical resources, is also an actual discussion topic in state of the art. In [27], the effective use of flight-time constrained devices is investigated, maximizing the average data service to ground users following a fair resource allocation policy. The solution of the cooperative allocation problem proposed in [28] significantly improves the performance of several network parameters. In [29], the authors try to minimize the number of vehicle-mounted BSs required to guarantee wireless coverage for a group of distributed ground users. Similar work in [30] proposes a placement algorithm for vehicle-mounted BSs that maximizes the number of covered ground users using the minimum UAVs. In [31], authors investigate the UAV coverage problem and propose a multi-UAV coverage model based on energy-efficient communication. The work in [32,33] focuses on the application of a multi-layout multi-subpopulation genetic algorithm achieving significantly better performance results than the other meta-heuristic algorithms also considered to improve the coverage deployment of multi-UAV networks. An explicit definition of the minimum-energy paths between a predefined initial and final configuration of a quadrotor by solving an optimal control problem concerning the angular accelerations of rotors is detailed in [34]. Their solution yielded minimum-energy and fixed-energy paths for the aerial vehicle.

## 3. Problem Statement

**Figure 2:**
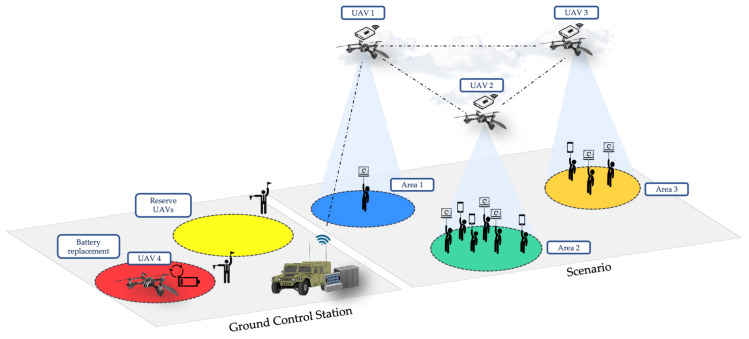
Multi-UAV system during a mission for three target areas (j=3) and four UAVs (i=4).

As it has just been mentioned, one of the main challenges in multi-UAV systems is to keep all the target geographic areas covered overtime by UAVs, since their battery life is limited (minutes) as compared to the typical mission timelines (hours). In order to face this problem, our approach is to use a fleet with the number of UAVs that are required to cover the whole scenario, and then maintain extra UAVs in a backup pool to serve as replacement units (as it can be seen in Figure 2). Once a replacement has been scheduled by the GCS, a fully recharged UAV enters the scenario while the replaced UAV goes back home to substitute its empty battery and be therefore ready to be changed by the next active UAV that requires a replacement. However, this procedure of identifying the minimum number of necessary (extra) UAVs and scheduling UAV replacements in the appropriate moment (to guarantee a minimum level of service availability) resembles a sophisticated approach and is the main problem that is treated in this paper.

**Figure 3:**
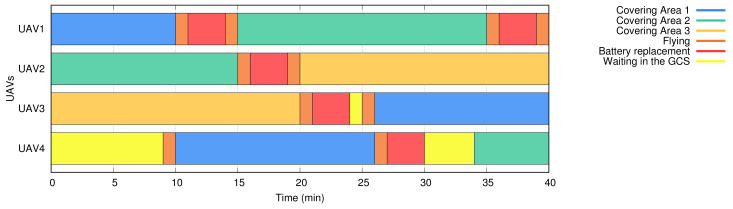
Multi-UAV system states during a mission for three target areas (j=3) and four UAVs (i=4).

Figure 2 depicts the reference scenario considered in our analysis. In this scenario, different colors are used to represent different geographic areas, which encompass the target areas where UAVs are intended to provide network coverage to end-users, the geographic location where UAVs are directed for a battery replacement, and the specific area where the backup pool of UAVs is kept for subsequent use. These color patterns have been reproduced in Figure 3 to classify not only what task the UAVs are doing at a certain moment but also to show which UAVs are covering the target areas at any given time. The following subsection faces the practical UAV replacement problem from an optimization viewpoint stating a simplified and manageable procedure, checking its complexity, and solving it through different approaches (optimal brute force algorithm, heuristic algorithm).

### Complexity Analysis

In this subsection, we prove that a simplified version of the proposed problem maps to an NP-hard problem (bin-packing problem [35] in this particular case) so that we are able to state its complexity. We denote a UAV using the index *i* and the target areas using the index *j*. Each UAV *i* has $C_{B_{i}}\left(t\right)$ battery level at instant *t* and the UAV might be in four different states: (i) battery replacement state (landed in the GCS), (ii) flying state (towards the GCS, or towards a region where it is intended to provide a network service), (iii) covering a region, or (iv) waiting in the reserve UAVs area to replace an active UAV. These four states can be appreciated in Figure 3. This diagram represents a hypothetical scenario with three regions (j=3) and 4 UAVs (i=4), also indicating when the replacements take place to guarantee system availability over time. Note that a region/area indicates where a UAV has to fly. In the case (e.g., the number of users, a high volume of traffic) that two UAVs have to be geographically near, we consider two different areas.

This problem statement must guarantee each region *j* is always covered by a UAV, i.e.,

(1) $$\sum_{i}x_{i,j}\left(t\right)=1,\;\forall j,\forall t$$

with $x_{i,j}\left(t\right)=1$ whenever UAV *i* covers region *j*. Moreover, at any time *t*, the battery level of UAV *i* has to stay above a safe threshold $a_{j}$ (e.g., 20%) for each region, so as to ensure the flight back to the GCS:

(2) $$\sum_{j}a_{j}x_{i,j}\left(t\right)\leq C_{B_{i}}\left(t\right)y_{i}\left(t\right),\;\forall i,\;\forall t,$$

here $y_{i}\left(t\right)=1$ whenever UAV *i* is not in the GCS.

Additionally, battery levels keep on decreasing while UAV is covering a region. Otherwise, we consider its battery levels is set to 100% once it has returned to the GCS, and the operator has replaced the battery:

(3) $$C_{B_{i}}\left(t+1\right)=\left(C_{B_{i}}\left(t\right)-c\right)y_{i}\left(t\right)+R_{T_{d},T_{r}}\left(1-y_{i}\left(t\right)\right),\;\forall i,\;\forall t.$$

with *c* the battery consumption, and $R_{T_{d},T_{r}}$ the average battery charge ratio during the time spent in returning to the GCS $T_{d}$, and the operator replacement task $T_{r}$.

$T_{d}$ remains constant in this simplified version of the problem, no matter how far a UAV *i* is from the region *j* it was covering, to the GCS.

The main goal of this problem is to minimize the number of active UAVs over time:

(4) $$\min\sum_{i,t}y_{i}\left(t\right).$$

This optimization problem with objective function (4), and constraints (1) and (2), maps to the bin-packing problem. Notice that this simplified problem has as bins the drones and as items the areas. Battery levels $C_{B_{i}}\left(t\right)$ are just the bin capacities (Note that having time-dependent variables corresponds to having *t* repeated in such variables multiple times, i.e., with $t=\left\{1,2\right\}$, $y_{i}\left(t\right)$ is expressed as two different variables $y_{i,1}$ and $y_{i,2}$.), and the battery threshold of each region *j* becomes the items’ weights. Thus, constraint (2) is just the bin-packing restriction that prevents exceeding bin capacities. Furthermore, constraint (1) imposes that all items (our regions *j*) are fitted inside a bin.

Without considering (2), we already have an instance of the bin-packing problem. Since this makes some instances of our problem being NP-hard, our reduced problem automatically becomes NP-hard. Then, the next step is to generate a heuristic algorithm that will provide a sub-optimal solution. At the same time, it is required to develop a methodology that will enable the algorithm evaluation.

## 4. Methodology

This section describes the different elements depicted in Figure 1 and explains the steps to be followed by the mission planner to provide uninterrupted network services. It first describes the parameters that UAVs must report to the GCS in order to serve as input for the scheduler algorithms. Then, it details the diverse assumptions taken for system modeling, that enable simulations to evaluate the preliminary proposals. Later, it presents the metrics to assess the performance of the proposed solutions. Finally, it describes the different strategies used in this article to schedule UAVs replacements.

### 4.1. Reported Parameters

Current UAV systems regularly report to their control station their location (GPS coordinates if the UAV incorporates this type of navigation) and the remaining battery. However, this knowledge may not be enough to have a holistic view of the UAV network which enables the scheduler algorithm to satisfy the objective function (4) of minimizing the number of required UAVs to provide guaranteed service availability. This is the list of parameters periodically reported by the UAVs to the GCS that enable the calculation of essential inputs for the scheduler algorithms that will be defined later to make the appropriate replacement scheduling:

- GPS coordinates: longitude, latitude, and altitude enable the calculation of the distance between each UAV and the GCS. Consequently, taking into account the cruising speed of the UAV, it is possible to estimate the time, and also the required battery, needed to complete the replacement procedure.
- Remaining battery: the current value of the available battery, in combination with the historical battery values (last *n* values), allows calculating the average energy consumption. With these values, an approximation of the UAVs’ lifetime can be determined.
- Network neighbors: the neighboring nodes enable us to generate a graph that represents the UAV network. With the GPS position and the theoretical wireless range, an overview of the network topology can be obtained. However, in certain circumstances, such as several packet collisions or high interferences, having a UAV nearby does not guarantee to have a proper communication channel established. Assuming that communications are bidirectional, for a network link to exist between two UAVs, both have to report each other to the controller, i.e., $UAV_{A}$ reports $UAV_{B}$ and $UAV_{B}$ reports $UAV_{A}$. This functionality is deployed in the UAV payload equipment.
- Number of connected users: if a UAV acts as a BS or AP (it can also act as relay, video transmitter, telemetry/sensor transmitter), it must report the number of users that it is serving. This way, we can better determine the impact that a failure (disconnection) of this particular UAV causes in the network.

### 4.2. Assumptions

Using a discrete-time model and in order to provide a reasonable implementation of the UAV replacement strategies, it is required to make some assumptions (simplifications):

1. The UAVs in the fleet are all the same model. It also implies that all batteries have the same dimensions and therefore have the same capacity/duration. This approach is reasonable since handling UAVs that use the same battery model reduces the number of these in the GCS and also simplifies the battery exchange procedure.
2. As long as there is a topological path existing between two nodes, it is assumed that the network route is possible and it is configured, i.e., no time is needed to configure different routes when topology modifications happen. Unquestionably, the routing protocol used in the network may eventually affect the system but under normal circumstances, the convergence time is negligible [36].
3. The chosen path between two network nodes (UAVs or network users) is the shortest path based on the number of hops. A priori, this decision makes sense since taking the shortest path minimizes the delay (and actually reducing the delay is one of the main objectives of 5G). However, if the network has several users distributed heterogeneously, using different paths may be interesting to balance the network load and avoid packet collisions.
4. When a UAV is in flight, any non-flight related energy consumption (for instance due to wireless transmissions) is negligible [37]. Furthermore, UAVs usually incorporate two batteries [38]. The primary battery is in charge of supplying the flying engines while the secondary supplies the payload equipment. The secondary battery enables a static mode of operation and, in particular situations, UAVs may land to extend the life of the provided network service, stopping the flying engines while keeping the payload powered with its own battery [21]. Our laboratory experiments with the secondary battery (3.7 V and 3800 mAh) result in more than 2 h of duration, so the battery limiting the UAV operation is the primary one in any case. Moreover, the battery consumption model is linear and the same in all the UAVs (it does not depend on the flight conditions).
5. Because the price of batteries is remarkably lower than the cost of UAVs, it is assumed that the number of available batteries cells is huge. This way, the GCS never runs out of charged batteries. Batteries can also be recharged during the mission, and some of them could be reused.
6. UAV payloads have enough computing capacity, consequently not saturated under any conditions. In our previous work [38], we have carried out experiments using Raspberry Pi 3B single board computers to prove their correct functioning.

Although all these assumptions may affect the results of the simulations, the primary purpose of this article is not to achieve accurate results but to verify that it is worth using a replacement scheduler algorithm to manage moderately large UAV fleets. Once this hypothesis is demonstrated, the progressive replacement of each simplification opens interesting future work for the evaluation of more realistic results.

### 4.3. Performance Metric

The average number of users connected (over time) is used as the metric to evaluate the performance of UAV replacement strategies. Each sampling period (e.g., 5 s in our simulations), this metric is examined in order to calculate the percentage of end-users connected to the GCS, which is in charge of providing Internet connectivity (i.e., the number of end-users that through a path established across the UAVs network are actually connected to the GCS). The average value of all the partial results during the simulation time will be used as performance metric.

### 4.4. Scheduler Algorithm Proposals

The following subsection outlines the strategies that have been taken into account when performing the simulations. Obtaining the optimal solution and defining a heuristic algorithm is part of the optimization process. The optimal solution will not predictably serve for large scenarios (in a reasonable time), but it will validate the heuristic algorithm in small scenarios for its future application in real environments. A summary of the parameters that describe the proposed UAV replacement strategies is shown in Table 1.

#### 4.4.1. Optimal Algorithm

To find the optimal UAV scheduling strategy that minimizes the number of UAVs used to cover a certain analysis region and a given number of users, the a brute-force algorithm has been proposed. This algorithm is an evolution of the strategy developed by us and presented in [8], which has incorporated positioning information, a number of users, and specific parameters related to the displacement between the GCS and the regions to be covered (i.e., landing time, take off time, and cruising speed of UAVs). In this regard, the proposed algorithm can be seen as an evolution of the approach addressed in [8]. The UAV scheduling strategy is explained in Algorithm 1, and its operation can be summarized in the following three stages.
**Algorithm 1:** Optimal UAV battery replacement strategy.
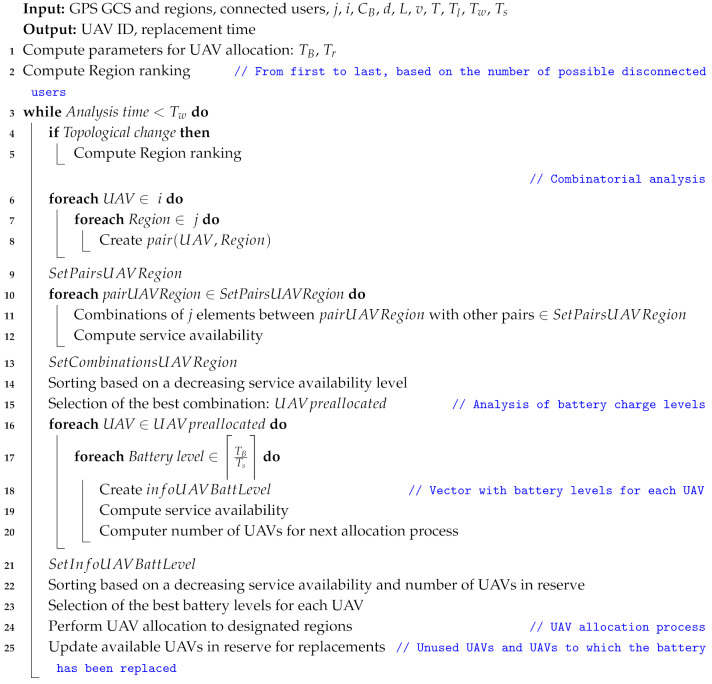

**Figure 4:**
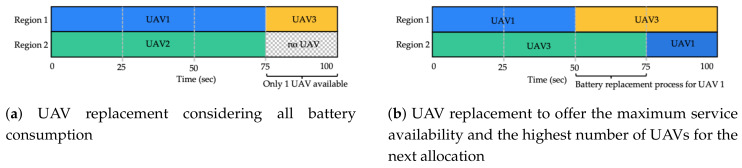
Differences between the analysed scheduling procedures. Example for j=2 and i=3 (2 UAVs in services and 1 UAV for replacement).

- Computation of parameters for UAV allocation. This procedure consists of calculating the lifetime of each UAV (i.e., the battery lifetime to exclusively provide the service in the designated region) and its corresponding replacement time, considering the information about the locations of the GCS and the service regions (GPS coordinates) as well as the parameters *v*, $T_{o}$ and $T_{l}$. In this step, a priority level or ranking is also assigned to each region according to the number of users that can be affected (disconnected) directly or indirectly if the UAV allocated to that region, and acting as an AP, suffers a failure. Thus, a higher priority level corresponds to a region that, if is not covered (no UAV allocated), it produces a higher number of disconnected users directly or indirectly (AP in that location with a link or links to other locations). This information is used in the process of allocation of UAVs to each region (next step), and ensures that fewer users are affected if i<j at a given time.
- Optimal distribution of UAVs to cover the service regions. Through a brute-force analysis, all possible combinations of available UAVs to cover the different service regions are explored. The best distribution (combination) of UAVs, which consists of those whose characteristics (battery duration) allow for the highest service availability time, is systematically selected. If necessary, a detailed example of the combinatorial analysis of UAVs to cover services is described in Table 4 in [8].
- Analysis of the percentage of battery charge to perform the replacement. With the information from the previous step (UAV allocation per service region), the algorithm analyses the optimal charge level for each UAV in which the corresponding replacement must be performed. This procedure is carried out by means of an exhaustive exploration of each level of charge for every UAV, and seeks to guarantee the highest service availability time and efficient use of the available resources (minimization of the number of UAVs for replacements). In a traditional approach, as shown in Figure 4a, replacement is performed when the battery capacity reaches its minimum threshold ($T_{B}=75$ s in the example). Although this procedure allows the full capacity of the battery to be used, the simultaneous discharge of several or all UAVs may cause a greater demand of resources (UAVs) for the subsequent allocations (in the worst case i=j) and unavailability of one or several regions if there are no UAVs available for replacements. On the contrary, a desynchronization in the replacement time, as shown in Figure 4b, allows not only a greater availability of services but also minimization of the number of UAVs in the system. In the example presented in Figure 4a, four UAVs are required (two UAVs in services and two in the reserve) to guarantee a service availability equal to 100%, whereas in Figure 4b, only three UAVs are necessary to reach the same availability level. Once the algorithm has determined the charge levels for a replacement that allow the maximum service availability and the maximum number of UAVs available for the next allocation, these UAVs are allocated to their corresponding regions. The allocation process continues iteratively (i.e., execution of step two and step three) until reaching the maximum time horizon $T_{w}$, as shown in the example in Figure 4b with $T_{w}=100$ s.

**Figure 5:**
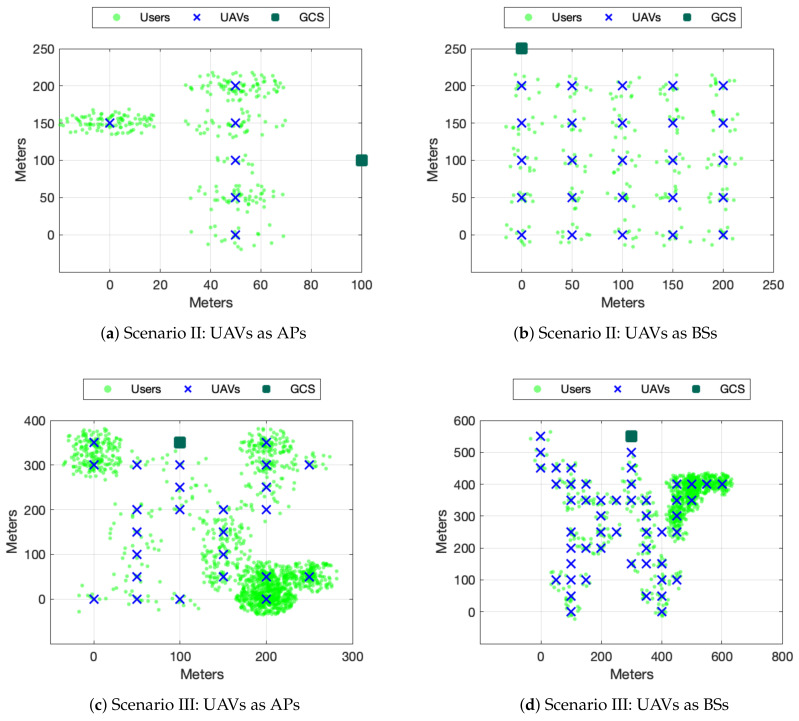
Proposed scenarios for algorithm performance evaluation.

The proposed optimal strategy is an offline exhaustive search mechanism whose complexity is given by (5)

(5) $$f\left(i,j,T_{B},T_{s}\right)=C\left(i\times j\;,j\right)+{\left(\left\lceil \frac{T_{B}}{T_{s}}\right\rceil \right)}^{j}$$

where the first term represents the combinatorial analysis for the allocation of UAVs and the second term corresponds to the analysis of the charge levels for replacements. Both terms in (5) are non-polynomial, the first term is the dominant and, according to the Big-O classification [39], the order of growth of the algorithm is O(C(ixj,j)), i.e., non-polynomial. Based on preliminary tests we can report that if the UAVs have the same characteristics (i.e., equal battery capacity) the analysis in step three (exploration of the charge levels) is only necessary for the first allocation process, because desynchronization is maintained all other allocations, as shown in Figure 5. While this mechanism can partially reduce the complexity of the algorithm, obtaining an optimal solution using exhaustive search limits it to real-time applications and reveals the drawbacks in selecting the number of regions and UAVs (at most i=12 and j=6). In this regard, this strategy can be used in planning stages to estimate the number of UAVs needed for a mission, such as an emergency or rescue scenario. However, in these cases a suitable alternative is a strategy described in [8], because it is a more generic and less complex approach.

Therefore, the hardness of the problem analysed in Section 3 (NP-Hard) and the complexity of the optimal solution shown in (5) (exponential) demonstrate the need for less complex heuristic mechanisms that can be used in real-time implementations. These strategies are described in the following sections and represent the major contributions of this paper.

#### 4.4.2. BETA: Betweenness Centrality Heuristic Algorithm

Heuristic algorithms are employed to solve optimization problems that are out of scope in reasonable times by optimal algorithms. In this particular case, it is also essential that this heuristic algorithm has a fast execution time because it must be run in real-time. BETA schedules the replacements based on the relevance of each participant within the network. To determine the relevance of an area in a network scenario, we apply graph theory fundamentals. Each area/UAV (an area is covered by a UAV) would correspond to the graph vertices (also called nodes), while the links among UAVs correspond to the graph edges (also called links or lines). One of the most well-known metrics to identify which are the most significant vertices in a graph is centrality, more specifically the betweenness centrality, which resembles the number of times a vertice acts as a connection along the shortest path between two other nodes. However, in the proposed multi UAV networks, nodes do not communicate with other nodes randomly since they do it with those that have Internet connectivity to the public network (either the GCS or a 5G-enabled UAV), as this provides the ground users with Internet connectivity.

To formulate this custom metric, we have divided the graph into two sub-graphs: (i) sub-graph which is composed by those UAVs that do not have Internet connectivity and (ii) sub-graph which is formed not only by the GCS, but also by the UAVs that may eventually have connectivity to the core network. Therefore, due to these specifications, the centrality metric has been calculated in the following way:

(6) $$g^{\prime }\left(v\right)=\sum_{\begin{array}{c} s\neq v\\ s\in A\\ t\in B \end{array}}\left(\frac{\sigma_{st}\left(v\right)}{\sigma_{st}}U_{s}\right),v\in A$$

where $\sigma_{st}\left(v\right)$ is the number of shortest paths from UAV *s* to UAV *t* that traverse UAV *v*, and $\sigma_{st}$ the total number of shortest paths from UAV *s* to UAV *t*. $U_{s}$ is the number of users connected to UAV $U_{s}$. The amount of users is crucial since if there are no users connected to UAV $U_{s}$, there is no impact on the network. This statement (6) (which is quite versatile) despite being designed for FANETs is also suitable for BS scenarios (where UAVs are directly connected to the core network).

A ranking was computed using the $g^{\prime }\left(v\right)$ metric as input. In case two UAVs have the same value $g^{\prime }\left(v\right)$ the one closer to the GCS will be above the other in the ranking since this will minimize the total replacement procedure time, and the replaced UAV will be active sooner to perform another UAV replacement. Now it is possible to assume which scheduling strategy to follow. BETA attends the following strategy (it can be appreciated in Algorithm 2): (i) if there is any topological change or the ground users move around the scenario, the algorithm must compute the ranking again; (ii) whenever there is a UAV available in the reserve, the algorithm schedules a replacement to the UAV with less remaining battery. However, this replacement takes place only if it does not affect UAVs that have a higher position in the ranking, i.e., that means that the remaining lifetime of the top UAVs is shorter than the time needed to make a UAV available again (after flying towards the GCS and battery replacement). In the case that this UAV replacement cannot be performed, the same analysis is repeated for the next UAV with the lower battery until the algorithm finds a UAV to make the replacement. For this algorithm to work correctly, it has to be executed periodically. In our case BETA runs every 5 s which coincides with the sampling period.
**Algorithm 2:** UAV replacement methodology.
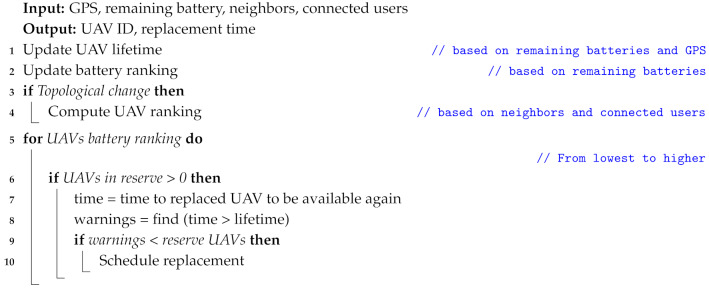


## 5. Simulation Details and Results

In order to validate these algorithms we have used different scenarios with different properties that will be discussed in this section. The following subsections detail *(i)* the simulation parameters and the justification of their selection, *(ii)* the ground-truth solutions with which the BETA algorithm is also compared, *(iii)* the simulation setup, and finally *(iv)* the simulated scenarios in combination with achieved results.

### 5.1. Simulation Parameters

This subsection details the parameters that have been taken into account to carry out the simulations and the selection criteria. This data, together with the related notation, can be seen in Table 1 and Table 2.

The time needed to perform a battery replacement is based on [15]. The battery capacity is based on the Parrot Bebop 2 specifications [7] (It is chosen because we have performed several tests using this model, and it is the selected unit in the technical validations we have worked previously [38,40,41], since it has demonstrated that it is able to carry a single board computer onboard like a Raspberry Pi for a reasonable time without problems and a reasonable cost). To calculate the device consumption, we have assumed that the UAV flies for 20 min (also specified in the technical characteristics). For WiFi range and although the standards state that the range is quite large, in practice, we have found that the WiFi range is relatively short for an acceptable received signal level [42]. The cruising speed has been calculated based on its maximum speed (also on technical specifications). Meanwhile, takeoff and landing times have also been calculated by our own measurements since we have not found accurate information. The simulations are iterated assuming a fixed number of areas to be served (and obviously one UAV per area) and then increasing the number of replacement UAVs (starting by 0 and increasing until the number of UAVs in reserve equals the number of UAVs in the scenario, which would mean doubling the size of the fleet).

### 5.2. Ground-Truth Solutions

To provide context to the BETA and optimal algorithms performance, they will both be compared with two alternative solutions (with smaller complexity). The primary purpose of the article is not to measure how far the heuristic solution is from the optimal but to highlight that the use of this type of solution is worthwhile and under which conditions and in which scenarios. In order to do that, the four scheduling techniques will be compared (BETA, optimal, baseline and simple scheduling) and different conclusions will be obtained

#### 5.2.1. Baseline

This is the simplest strategy. UAVs are assumed to periodically send their current battery level and GPS position, however, no further calculations are made from the GCS. When an active UAV reaches a minimum battery threshold, i.e., only the required battery to return to the GCS plus a safety threshold, e.g., 20%, a replacement is scheduled (if UAVs are available), i.e., the drained UAV flights to the GCS, and at that moment (when the drained UAV starts flying to the GCS), a fresh UAV takes off and flies to the uncovered target area to provide the service. If no fresh UAVs are available, there will be no service in that area until a UAV is ready to go and cover it again. The lack of intelligence in this baseline solution prevents us from reaching 100% service provisioning in any case because even with infinite UAVs to serve as fresh replacements there will always be a gap without network service corresponding to the time that passes since the drained UAV leaves the stage towards the GCS until the moment the new UAV enters the stage and starts operating.

#### 5.2.2. Simple Scheduling

This strategy is inspired by [15]. UAVs are also assumed to send their current battery level and GPS periodically. However, in this case, the controller is required to estimate a battery threshold (based on battery reports and other parameters such as the UAV speed and the takeoff and landing times) that includes not only the minimum battery needed to return to GCS but also includes the time needed for the fresh UAV (in case there are available units) to reach the target area. That way the new UAV will start serving the area just after the old one leaves and predictably, if there are enough UAVs in reserve, all the areas can be covered for the whole mission time (or at least a high percentage of the time). In case the active UAV reaches the threshold and there is no fresh UAV to perform the replacement, the UAV can still continue providing service until it reaches the battery level needed to reach the GCS enlarging the service time.

### 5.3. Simulation Setup

A Matlab (Matlab R2017b) event-based simulator achieves all the paper results. To calculate the BETA, simple scheduling, and baseline solutions in all the scenarios, a computer equipped with a 2.6 GHz Intel Core i5 processor and 8 GB RAM was used. Meanwhile, the optimal algorithm has been run on a computer equipped with a 3.33 GHz x 12 cores Intel Core i7 Extreme processor and 24 GB RAM. If the reader is interested in reproducing the experiment, the code is available in this repository [43].

### 5.4. Validation Scenarios

#### 5.4.1. Scenario I: Proof of Concept

**Figure 6:**
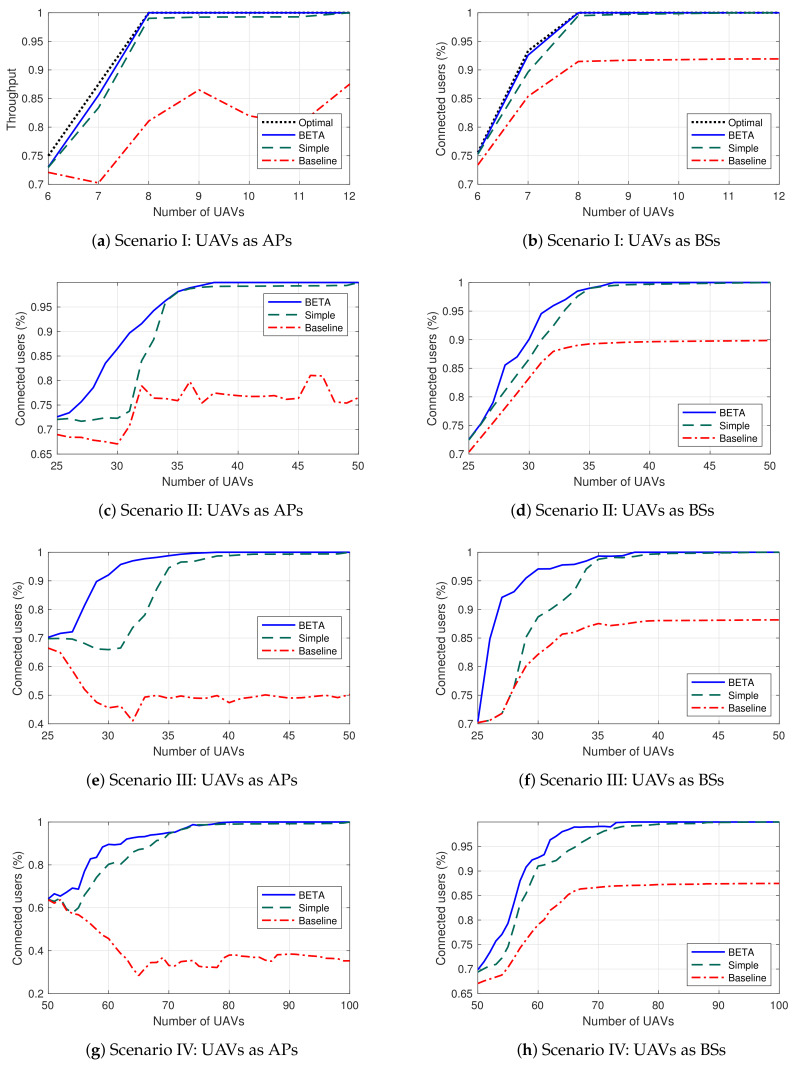
Average number of users connected in different scenarios increasing the fleet size.

To start the analysis, we have defined a basic scenario (it can be appreciated in Figure 5a) as a proof of concept. In this stage, there are a total of 6 coverage areas and 300 ground users heterogeneously distributed. In a scene with these reduced dimensions, it is possible (in terms of reasonable computation time) to run the algorithm that provides the optimal solution so it will be possible to compare all the alternatives. Figure 6a depicts the average connected users for the four algorithms when the UAVs act as a FANET which means that UAVs onboard commodity WiFi equipment and use the created UAV WiFi adhoc network itself to connect to the GCS (which in turn provides the Internet connectivity). Therefore if one of the UAVs that are geographically closer to the GCS (and hence connecting part of the topology to the GCS) runs out of battery and there is no possible UAV replacement, some parts of the network may get disconnected even though the rest of UAVs may be successfully covering other target areas. Following this logic, whenever there is a failure in the backbone UAV, the system gets completely divided. On the other hand, Figure 6b depicts the average connected users for the four algorithms when UAVs act as BSs (they are directly connected to the public network without the need of a hop by hop network like a FANET). These scenarios are usually employed in massified events where the existing cellular network is operating correctly, but may be insufficient. As expected, these results are better than the FANETs results since each UAV is only responsible for its own end users. However these on-boarded BS solutions are usually more expensive and it is not always viable (when the infrastructure does not exist or is temporarily damaged for instance).

Figure 6a shows that both BETA and the simple scheduling strategies perform similarly and are close to the optimal solution. To reach 100% of connected users with the simple scheduling approach, it is required to double the UAV fleet (12 UAVs) but in any case in reduced scenarios, the simple scheduling solution is enough to provide an adequate service. On the other hand, the baseline algorithm provides erratic and unintuitive results considering that the performance decreases as the fleet increases. This phenomenon happens because although the time that UAVs are covering the target areas is higher, the network is disconnected for longer, i.e., having more UAVs does not guarantee overall connectivity if the backbone UAV is not working. If there are no reserve UAVs in reserve (the fleet size is equal to the number of target areas), the return and battery replacement process (of all the UAVs in the scenario) is almost synchronized (and operate simultaneously). However, if there are some UAVs in reserve, this process may be unsynchronized. For this reason, the baseline results decrease and, in consequence, are worse and inconstant.

The results in Figure 6b (UAVs acting as BSs) are better as we commented and again BETA and simple scheduling strategies are close to the optimal solution. The baseline solution performance improves in this case, as the size of the fleet increases. All the strategies in fact stabilize with a fleet of 8 UAVs, two in reserve (fleet 25% oversize), and both BETA and simple scheduling achieve acceptable values.

In scenarios with reduced dimensions this 25% of fleet oversize (having two UAVs in reserve) seems quite reasonable. However, in a scenario with numerous areas, e.g., 25 areas, 50 areas, this oversize may imply a rather expensive operation. It is then important to validate the solutions in much bigger scenarios and see the performance of the algorithms there.

#### 5.4.2. Scenario II: Grid

Figure 5b shows a scenario with 25 coverage areas and 250 ground users homogeneously distributed, i.e., ten ground users per area. We have selected a grid topology which is fail-tolerant since there are multiple alternative paths to reach the GCS from each area. Moreover, all the areas have the same number of users, which makes the difference in the UAV ranking insignificant in the FANET scenario and almost nonexistent in the BSs scenario.

Figure 6c shows that the performance of the heuristic strategy is better than the simple scheduling solution (when the fleet is formed by 30 UAVs, 5 UAVs in reserve, the results improve by more than 10%). Both strategies reach acceptable levels from 35 UAVs fleet. The heuristic solution reaches 100% of users connected with a 38 UAVs fleet while the simple scheduling solution, as in the first scenario, needs to double the fleet size to reach 100% of connected users. On the other hand, the baseline solution has similar behavior to the previous scenario. This outcome highlights that if no strategy (however simple) is used to schedule the UAV replacements, the results can be harmful, and even over-dimensioning the resources does not guarantee favorable results.

Figure 6d presents the results of UAVs acting as BSs. In this case, the heuristic algorithm behaves similarly to the simple scheduling solution. The heuristic algorithm schedules the UAV replacements based on $g^{\prime }(v)$ metric (6), which is determined using graph theory. In this scenario, the nodes representing the UAV network have the same $g^{\prime }\left(v\right)$ since they are all directly connected to the infrastructure and provide connectivity to the same ground users; therefore, all UAVs connect the same number of users to the network. For this reason, scheduling the UAV replacements using the heuristic strategy has no advantage other than that they are performed as soon as there is an available fresh UAV. This phenomenon reveals that the heuristic solution makes the difference in scenarios where UAVs have different relevance within the network.

#### 5.4.3. Scenario III and Scenario IV: Tree

Finally, we have designed two tree type scenarios with the users distributed very heterogeneously. This type of scheme makes some UAVs much more relevant, and scheduling replacements effectively seems to have a substantial impact on the final performance. The first scenario has 25 coverage areas and 300 ground users. The second scenario has 50 coverage areas, 500 ground users. The areas and user distribution can be appreciated in Figure 5c,d.

Figure 6e reveals that the difference between the BETA solution and the simple scheduling solution is significant in these scenarios. For a 30 UAVs fleet (5 UAVs in reserve), we achieve a 20% improvement by using the heuristic scheduler, which is an important variation when providing a network service. The heuristic strategy obtains 100% of users connected from 36 UAVs. As in previous scenarios, the baseline solution produces insufficient results. It is interesting to observe that as the UAV fleet increases (which have high economic cost), the users are not connected longer.

Similarly, when UAVs act as BSs, Figure 6f, we obtain better results using the heuristic strategy. This variation is because, in this scenario, the ground users are heterogeneously distributed, and consequently, UAVs have different $g^{\prime }\left(v\right)$ since they connect diverse numbers of ground users, which implies that performing the correct replacement has more impact.

The conclusions of scenario IV are similar to the ones of scenario III, although the performance (Figure 6g,h) is worse because of the greater complexity of the UAV network topology and the greater failure possibility.

### 5.5. Comparison of the UAV Replacement Strategies

Once the results have been obtained (Figure 6) and discussed (Section 5.4), in this section we will perform a comparison of the results by computing: *(i)* for scenario I, the distance from the optimal solution (Opt) to the suboptimal or approximate solutions (SubOpt), in order to verify the quality of the results, and *(ii)* for the succeeding scenarios, the difference between the simple scheduling and baseline approaches against the Heuristic strategy. To this end, the criterion of approximation ratio (ρ) has been used [23]. This parameter, in the context of the proposal, estimates how many times lower the approximate solution is as compared to the exact or optimal result; it is defined as the average value of the ratio between the suboptimal and optimal solutions, as shown in

(7) $$\rho =\frac{1}{i}\sum_{i}\frac{SubOpt_{i}}{Opt_{i}},$$

where $SubOpt_{i}$ and $Opt_{i}$ are the results for all the variation of UAVs in reserve (from zero to fleet size) for the optimal and suboptimal strategies, respectively. The ρ factor ranges from 0 (whether Opt and SubOpt have completely different values) to 1 (if Opt and SubOpt produce the same solution); an intermediate value (i.e., 0<ρ<1) represents the similarity or closeness factor to the optimal solution (SubOpt=ρxOpt) [23]. For a better understanding of the calculation of this parameter, (8) presents an example for the simple scheduling approach of Scenario I when UAVs act as APs (Figure 6a).

(8) $$\rho =\frac{1}{7}\times \left(\frac{72.95}{74.99}+\frac{83.38}{87.51}+\frac{99.02}{100}+\frac{99.24}{100}+\frac{99.27}{100}+\frac{99.28}{100}+\frac{100}{100}\right)=0.98.$$

**Figure 7:**
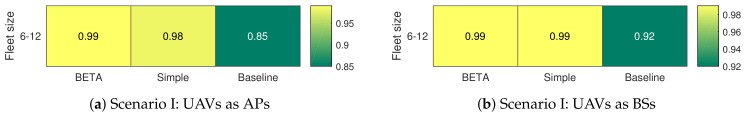
Approximation ratio ρ: optimal strategy vs. heuristic strategies for Scenario I.

**Figure 8:**
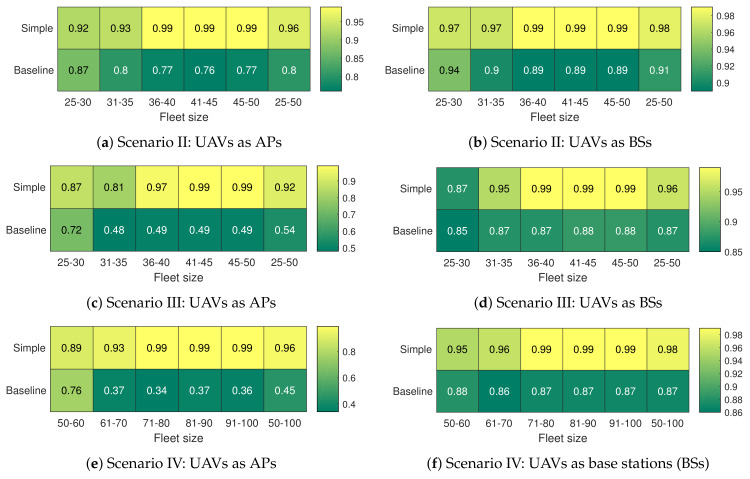
Approximation ratio ρ: betweenness centrality heuristic algorithm (BETA) vs. other suboptimal strategies.

The result of (8) shows that the suboptimal solution (simple scheduling approach) is similar to the optimal solution in a factor equal to 0.98 (98% similarity between solutions). The rest of the ρ factors for Scenario I (Figure 6a,b) are summarized in Figure 7, while the ρ values for other scenarios are presented in Figure 8. The comparison between the optimal solution and the approximate solutions in Scenario I, based on ρ factor, reveals that all the proposed strategies produce not only near-optimal solutions, but also a stable performance (i.e., high-quality feasible solutions). In all cases, as illustrated in Figure 6a,b and then corroborated in Figure 7, the approximate algorithms (BETA, simple scheduling and baseline) generate solutions very close to the optimal, even with 99% similarity to the exact result (1% of error), which is achieved by the BETA approach. Then, this strategy is used as a baseline to evaluate the performance of the other strategies (simple scheduling and baseline) for Scenario II, Scenario III, and Scenario IV. In summary, from the information analyzed in Section 5.4, the average number of connected users allows us to appreciate where one algorithm improves another, while the distance to the optimal solution computed in this section allows us to quantify this variation. To provide a higher level of detail, ρ has been analyzed by ranges depending on the fleet size (all the analysis has been carried out by increasing the number of UAVs gradually). In addition, this value has also been computed at a global level. The main reason is to analyze in which areas the solution improves and quantify it. Since from a particular value, the solutions provide similar results, computing this metric at a global level makes it difficult to recognize the areas of improvement.

It can be seen in Figure 8 that the central area of improvement is in the first two ranges, i.e., from 25 to 35 UAVs. This result is positive because, as expected, if there is a reasonable amount of UAVs (with their corresponding cost), a typical solution can perform adequately. However, in scenarios with limited resources using the heuristic strategy improves in all cases the simple scheduling solution.

Analyzing the above metrics, we can conclude that using strategies to make replacements is worthwhile. However, the heuristic strategy designed in this article is considerably aggressive since it schedules a replacement whenever UAVs are available. This strategy can result in the number of replacements skyrocketing over time, as well as the number of batteries to be used, which would bring a high economic cost. It should be noted that the price of a battery is much lower than that of a UAV but in any case it is not negligible.

**Figure 9:**
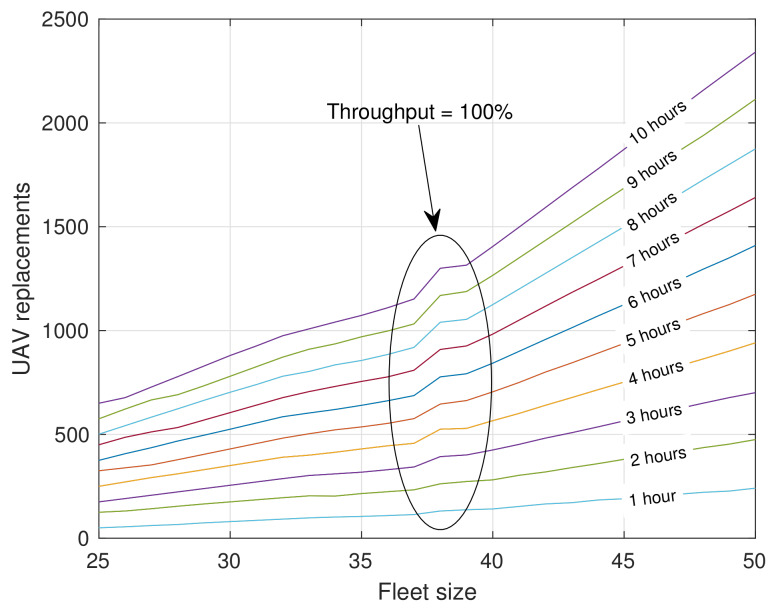
Number of UAV replacements using BETA in scenario III.

Figure 9 displays the number of UAV replacements as a function of the number of UAVs forming the fleet and the mission time for scenario III using the BETA strategy. Approximately 1200 replacements are needed to provide a service of 10 h and obtain 100% of users connected. Furthermore, due to the aggressive nature of BETA, the replacement grows exponentially after reaching a fleet size that guarantees 100% of connected users. In addition, it should be mentioned that the main advantage of small UAVs is that deployments are generally done very quickly and very flexibly, but as we have seen it is both economically and logistically difficult, to achieve reasonable solutions when service time largely exceeds battery lifetime. Other alternatives should be used in these cases like bigger UAVs with more battery capacity (or even using fuel), land the UAVs on the ground to improve their autonomy, or even deploying of a fixed infrastructure if service is expected to be maintained for a long.

## 6. Conclusions and Future Work

This article states the practical UAV replacement problem, where a multi-UAV network is expected to provide long-endurance network services (in the order of hours) using constrained devices with limited autonomy (in the order of minutes). It is verified that the optimal UAV scheduling to minimize the number of UAVs for replacements while providing a guaranteed service availability, is NP-hard and that its optimal solution has exponential complexity. In this regard, some heuristics approaches have been analyzed and evaluated.

Secondly, the article details a methodology, including the simulation environment and the parametrization required to perform a preliminary evaluation of these heuristic strategies. The simulator code is available in [43] to reproduce the experiment and evaluate upcoming future strategies.

The article also introduces BETA, a heuristic replacement strategy that performs the replacements as soon as possible based on the relevance of each UAV within the network. BETA is presented as an example in order to verify if it is worthwhile using a heuristic replacement approach or not. BETA is capable of running in real-time with a 99% similarity with the optimal solution in some simple scenarios (scenario I). In heterogeneous scenarios, BETA improves the basic solutions, achieving the most significant improvement in instances where the scenarios are heterogeneous, and the resources are limited. Furthermore, we conclude that it is far better to have a replacement strategy (no matter how simple it is) than having no strategy at all. BETA is compared with the optimal algorithm in order to evaluate the distance whenever possible and with other alternatives in some other scenarios and it has been possible to see that in some situations the advantages are not so relevant as in other ones.

The article opens several lines of future research, such as to be able to provide priority of replacements for UAVs serving users in emergency/disaster scenarios. The application of replacement strategies in disaster scenarios includes an uncertainty degree caused by several factors caused by moving UAVs while they are operating (that may change the topology), or extreme conditions that may force the engines and battery consumption. Some other futures lines include the combination of FANETs and BSs in the same scenario, to test UAVs models with different battery capacity, to model the energy consumption according to more realistic consumption patterns based on experimentation, or to schedule UAV replacements without making them through the GCS.

## Figures and Tables

**Table 1:** System parameters.

Parameter	Notation	Units/Coments
Number of regions	*j*	Integer number
Number of UAVs	*i*	Integer number
Location GCS	$P_{GCS}$	x,y coordinates
Location UAV${}_{i}$	$P_{UAV_{i}}$	x,y coordinates
Number of users per region	$u_{j}$	Integer number
Total number of users	*U*	Integer number
Battery replacement time	$T_{r}$	Time units, e.g., seconds
Battery capacity	$C_{B}$	Electric current per time units, e.g., mAh
Device consumption	*d*	Electric current, e.g., mA
Link distance	*L*	Length units, e.g., meters
UAV cruising speed	*v*	Speed units, e.g., meters/seconds
Take-off time	$T_{o}$	Time units, e.g., seconds
Landing time	$T_{l}$	Time units, e.g., seconds
Simulation time	$T_{w}$	Time units, e.g., seconds
Sampling time	$T_{s}$	Time units, e.g., seconds

**Table 2:** Simulation parameters.

Scenario	Parameters											
	j	i	U	$T_{r}$	$C_{B}$	d	L	v	$T_{o}$	$T_{l}$	$T_{w}$	$T_{s}$
I	6	6–12	300	180 s	2700 mAh	5670 mA	70 m	5 m/s	60 s	60 s	3600 s	5 s
II	25	25–50	250									
III	25	25–50	300									
IV	50	50–100	500									

## Abbreviations

The following abbreviations are used in this manuscript:

UAV	Unmanned Aerial Vehicle
5G	Fifth-generation cellular network
KPI	Key Performance Indicator
BS	Base Station
AP	Access Point
FANET	Flying Ad Hoc Network
GCS	Ground Control Station
SUAV	Small Unmanned Aerial Vehicle
GPS	Global Positioning System
BETA	Betweenness centrality heuristic algorithm
IoT	Internet of Things
LTE	Long Term Evolution
EPC	Evolved packet core
RAN	Radio access network
MEC	Multi-access edge computing
SUAV	Small Unmanned Aerial Vehicles
NP	Nondeterministic polynomial
RAM	Random Access Memory
